# Minimum spanning tree analysis of brain networks: A systematic review of network size effects, sensitivity for neuropsychiatric pathology, and disorder specificity

**DOI:** 10.1162/netn_a_00245

**Published:** 2022-06-01

**Authors:** N. Blomsma, B. de Rooy, F. Gerritse, R. van der Spek, P. Tewarie, A. Hillebrand, W. M. Otte, C. J. Stam, E. van Dellen

**Affiliations:** University Medical Center Utrecht, Department of Psychiatry, Brain Center, Utrecht, the Netherlands; Amsterdam UMC, Vrije Universiteit Amsterdam, Department of Neurology and Department of Clinical Neurophysiology and MEG center, Amsterdam Neuroscience, Amsterdam The Netherlands; University Medical Center Utrecht, Department of Child Neurology, Brain Center, Utrecht, the Netherlands; University Medical Center Utrecht, Department of Intensive Care Medicine, Brain Center, Utrecht, the Netherlands

**Keywords:** Minimum spanning tree, network neuroscience, transdiagnostic, multimodal, network size

## Abstract

Brain network characteristics’ potential to serve as a neurological and psychiatric pathology biomarker has been hampered by the so-called thresholding problem. The minimum spanning tree (MST) is increasingly applied to overcome this problem. It is yet unknown whether this approach leads to more consistent findings across studies and converging outcomes of either disease-specific biomarkers or transdiagnostic effects. We performed a systematic review on MST analysis in neurophysiological and neuroimaging studies (*N* = 43) to study consistency of MST metrics between different network sizes and assessed disease specificity and transdiagnostic sensitivity of MST metrics for neurological and psychiatric conditions. Analysis of data from control groups (12 studies) showed that MST leaf fraction but not diameter decreased with increasing network size. Studies showed a broad range in metric values, suggesting that specific processing pipelines affect MST topology. Contradicting findings remain in the inconclusive literature of MST brain network studies, but some trends were seen: (1) a more linelike organization characterizes neurodegenerative disorders across pathologies, and is associated with symptom severity and disease progression; (2) neurophysiological studies in epilepsy show frequency band specific MST alterations that normalize after successful treatment; and (3) less efficient MST topology in alpha band is found across disorders associated with attention impairments.

## Author Summary

The potential of brain network characteristics to serve as biomarker of neurological and psychiatric pathology has been hampered by the so-called thresholding problem. The minimum spanning tree (MST) is increasingly applied to overcome this problem. We performed a systematic review on MST analysis in neurophysiological and neuroimaging studies and assessed disease specificity and transdiagnostic sensitivity of MST metrics for neurological and psychiatric conditions. MST leaf fraction but not diameter decreased with increasing network size. Contradicting findings remain in the literature on MST brain network studies, but some trends were seen: (1) a more linelike organization characterizes neurodegenerative disorders; (2) in epilepsy there are frequency band specific MST alterations that normalize after successful treatment; and (3) less efficient MST topology is found across disorders associated with attention impairments.

## INTRODUCTION

A biomarker can be defined as a characteristic that is objectively measured and evaluated to indicate normal biologic processes, pathogenic processes, or pharmacologic responses to a therapeutic intervention (De Gruttola et al., 2001). In psychiatry, and to a lesser extent in neurology, clinical practice and therapeutic innovation lack biomarkers (First et al., 2018).

Disturbances in the organization of macroscale brain networks are increasingly recognized as a pathophysiological characteristic of brain disease (Bullmore & Sporns, 2009). A recurrent finding across neurological and psychiatric disorders is the loss of network efficiency or integration, and damage to hub regions (Bassett & Bullmore, 2009; Crossley et al., 2014; Stam & van Straaten, 2012). Brain network metrics may thus have the potential to serve as biomarkers that will aid the diagnostic process and guide treatment, provided that one or more reliable and reproducible indicators can be established (Douw et al., 2019; First et al., 2018). Thus far, however, different studies describing changes in brain networks for the same disorder have yielded contradictory results. These results can at least in part be explained by methodological issues (Fornito et al., 2013; Tijms et al., 2013; van den Heuvel et al., 2017; Van Diessen et al., 2013). In brain network research, one key issue is the definition of a ‘true’ connection or edge based on empirical data. Weighted connection estimates from inherently noisy data will introduce false positive connections in the network. Furthermore, comparing networks with differences in mean connection weights introduces possible bias since this influences graph measurements such as clustering coefficient and path length (van Wijk et al., 2010).

A frequently used solution is to threshold connection weights, but this introduces the so-called “thresholding problem”: the choice of a threshold is often arbitrary (van Wijk et al., 2010; Fornito et al., 2012; Stam et al., 2014; Zalesky et al., 2016). Fixed thresholds may lead to different connection densities across subjects that bias higher order graph characteristics, while fixed densities include noisy edges or discard true edges (Ercsey-Ravasz et al., 2013). Attempts to normalize data may thus inherently introduce bias to graph theoretical measures. The thresholding problem contributes to poor reproducibility and limited interpretability of results.

Stam and others put forward a theoretical solution, at least at the macroscale brain network analysis level, by reconstructing the minimum spanning tree (MST) (Stam et al., 2014). A tree is defined as a connected graph with a path between each pair of nodes, without forming any loops. A spanning tree is defined as a subgraph that includes all *N* nodes of the original graph and *N* − 1 links (*m*). When the sum of the weights of the links is minimized, this is called a MST of the connected weighted graph (Kruskal, 1956; Prim, 1957). Importantly, the MST will serve as the backbone of information flow in the network under conditions where link weights in the original graph show strong fluctuations (Van Mieghem & Van Langen, 2005). Advantages of this approach are that the MST of the weighted connectivity matrix is unique, provided that its weights are unique. MST connections may be binarized to avoid density effects, and the number of links in the MST is fixed (Stam et al., 2014). And, importantly, MST characteristics can be interpreted along the lines of conventional metrics that characterize network topology (Tewarie et al., 2015a). Tewarie and others further demonstrated in silico that MST analysis indeed is reliable and reproducible, in the sense that it is relatively insensitive to bias and noise in simulated connectivity data (Tewarie et al., 2015a). MST metrics were unaffected by changes in density and average connectivity of a network, and global MST metrics are even robust against substantial levels of noise in the input data.

Other, more data-driven thresholding approaches are also available, including efficiency cost optimization, proportional thresholding, and probabilistic thresholding (De Vico Fallani, Latora, & Chavez, 2017; van den Heuvel et al., 2017; Váša, Bullmore, & Patel, 2018). The MST has been proven to be theoretically and methodologically reliable for specific imaging modalities (van Dellen et al., 2018). Over the last few years, MST analysis has been applied to neuroimaging and neurophysiological data obtained with various acquisition techniques, such as magnetic resonance imaging (MRI), functional magnetic resonance imaging (fMRI), diffusion tensor imaging (DTI), electroencephalography (EEG), and magnetoencephalography (MEG).

Here, we performed a systematic literature review and critically assessed whether the use of MST analysis to characterize brain networks holds promise for establishing the reproducibility and reliability necessary for the development of brain network–based biomarkers for neurological and psychiatric disorders. First, we analyzed how MST metrics are affected by different imaging modalities, node, and link definitions by comparing MST characteristics in empirical studies of healthy controls.

Secondly, we assessed how results from different MST studies compare within and across categories of pathology, and attempted to interpret the changes in brain networks that occur in brain disease from a transdiagnostic perspective. After a systematic search for clinical MST studies, we categorized findings on (1) neurodevelopmental disorders, (2) adult psychiatric disorders, (3) neurodegenerative disorders, (4) multiple sclerosis, (5) epilepsy, and (6) other neuropsychiatric disorders.

## METHODS

### Search Term and Search Strategy

References were identified through searches of PubMed and EMBASE, using an array of terms covering brain connectivity in combination with MST. Exact search terms are specified in the Supporting Information. Furthermore, Google Scholar was used to search for articles from 2005 and onward citing the following key papers: Kruskal (1956), a seminal paper on graph theory; Stam et al. (2014), an extensive review on the methodological state of affairs in brain network research which has proposed the MST as a possible solution for various methodological issues; Tewarie et al. (2015a), a study showing the relevance and reliability of MST graph theoretical approach for studying brain networks (Kruskal, 1956; Stam et al., 2014; Tewarie et al., 2015a). For the paper by Kruskal, search results were further narrowed down by using the following search terms “brain OR neuronal OR cerebral.” Searches were conducted from inception to May 2020.

The resulting articles were reviewed for relevance on title and abstract by two independent raters (FG and NB and/or BdR). If the article was deemed potentially relevant, the full text was also reviewed. In case of uncertainty about whether an article was eligible for inclusion, a third rater (EvD) coreviewed the article and was then included or excluded by consensus.

Articles were included when they met the following criteria: published in English, assessing macroscale, whole-brain network topology through the use of one or more of these four imaging techniques that are broadly used in studies on neurological and psychiatric disorders: electroencephalography (EEG), magnetoencephalography (MEG), functional MRI (fMRI), DTI; conducted during resting-state and without intervention; having constructed a minimum spanning tree and reported at least one of the following MST measures: diameter, leaf fraction, leaf number, and kappa and tree hierarchy in a population with a neurological or psychiatric disorder. When available, numeric values for these measures were extracted from the original articles or Supporting Information.

### MST Metrics

**Figure 1.**
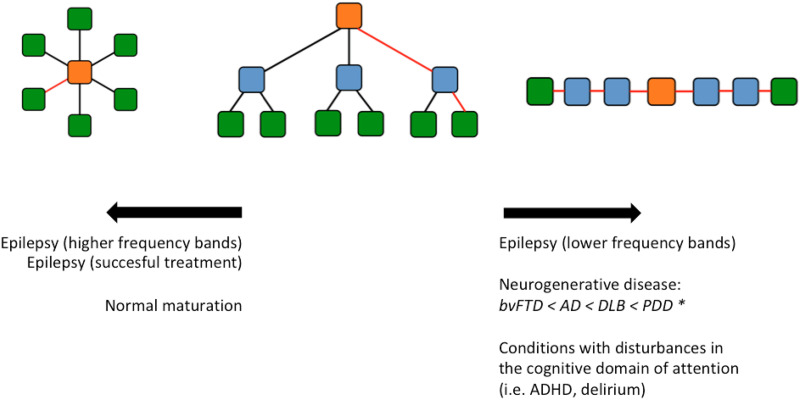
Schematic depiction of three different minimum spanning trees, with a starlike, intermediate and linelike configuration from left to right. The green nodes represent leaf nodes. Central nodes are depicted in orange. Diameter is depicted in red. Individual conditions and the correlated changes in network topology as described in the discussion section are displayed, with an arrow depicting the direction of the change. For neurodegenerative diseases conditions are displayed left to right from having the least shift toward a more linelike topology (bvFTD) to the most (PDD). AD, Alzheimer’s disease; bvFTD, behavioral variant of frontotemporal dementia; DLB, dementia with Lewy bodies; PDD, Parkinson’s disease dementia.

MST measures that were analyzed included diameter, leaf fraction, and kappa and tree hierarchy, which describe the integration and efficiency of the network. A description of these measurements is provided in Table 1, where Figure 1 provides a schematic overview of MST topologies and corresponding characteristics.

**Table 1.** Explanation of the MST measurements included in this review

**Symbol**	**Concept**	**Explanation**
D	Diameter	A measure of network efficiency and refers to the largest distance (in number of links) between any two nodes and is normalized for the total number of links: *D* = *d*/*M*, where M is the total number of links or maximum leaf number (*M* = *n* − 1, with *n* the number of nodes). An increase in diameter, means a decrease in global efficiency, whereas a low diameter indicates a more efficient information flow between brain regions.
LF	Leaf fraction	A measure of centrality and is based on the leaf number; the number of nodes with only one connection. The leaf fraction (L_f_) is the leaf number (L) divided by the maximum possible leaf number: *L*_f_ = *L*/*M*. This measure ranges between 2/*M*, which indicates a linelike topology and a maximum value of *M* = *n* − 1 (*n* = number of nodes), which indicates a star topology. A lower value of the leaf fraction indicates a less centralized network topology and a high leaf fraction means that communication depends strongly on hub nodes (i.e., nodes that play a central role in the network).
Th	Tree hierarchy	Quantifies the trade-off between large-scale integration in the MST and the overload of central nodes, calculated by Th = L/(2mBCmax) (Boersma et al., 2013), where BCmax stands for the maximum value of the betweenness centrality among all the nodes in the MST, and BC itself is computed as the fraction of shortest paths that go through a node. Note that nodal BC and BCmax were not considered macroscale network characteristics and therefore excluded from analysis in this review. To assure tree hierarchy ranges between 0 and 1, the denominator is multiplied by 2. Th ranges between 0 and 1, where Th approaches 0 if *L* = 2 (linelike topology) and *M* approaches infinity. For *L* = *M* (starlike topology), Th approaches 0.5. A network topology that optimizes a trade-off between integration and segregation is hypothesized when Th approaches 1 (Stam et al., 2014).
κ	Kappa	A measure of the broadness of the degree distribution or the heterogeneity of degrees and relates to the spread of information across the tree (Stam et al., 2014). A low value of kappa indicates a low number of highly connected nodes (hubs). High kappa values are especially seen in scale-free networks (Stam & van Straaten, 2012).

A small diameter combined with a high leaf fraction characterizes a more starlike, centralized network. In contrast, a large diameter with a low leaf fraction indicates a more linelike topology and decentralized network. A starlike network is characterized by short paths between the most remote nodes and thus facilitates efficient transfer of information across the network. However, the central node is burdened by a relatively large flow of information in such a network, possibly creating a greater chance of overload. Starlike networks will also be more vulnerable to targeted attacks to central hub nodes (Otte et al., 2015).

### Meta-Analysis

Due to limited available data, kappa and tree hierarchy were excluded from quantitative analysis. The MST variables diameter and leaf fraction were included in further quantitative analyses. First, we tested if MST variables were affected by network size. Mean values of MST metrics from healthy control groups were used for this analysis. Due to a limited number of data points, and the fact that some fMRI-based studies had considerably more nodes, we decided to use nonparametric linear regression method according to Siegel and others, to ensure robust regression (Siegel, Donner, & Engel, 2012). For EEG and MEG data, we followed the authors’ frequency bands.

Second, to analyze the network deviations in various brain disorders and interpret results from a transdiagnostic perspective, mean group effects on MST metrics were analyzed based on comparisons between clinical and control groups. We performed both fixed-effect and random-effects meta-analysis on the standardized difference of the mean estimates of mean leaf fraction per study and mean diameter per study. Studies were stratified by imaging modality. MEG/EEG studies were aggregated, but stratified for each frequency band. Heterogeneity was assessed using I^2 calculated based on Cochran’s Q. Analyses were performed using the meta package in R 3.6.1.

Finally, we provided a narrative review to evaluate if MST analysis holds promise for biomarker development of brain disease, where insufficient data were available to draw definitive conclusions on quantitative outcomes.

## RESULTS

The search resulted in 43 included studies in this review, 34 described a patient-control study design, and 9 studies included children. The 43 studies were further subdivided into papers on neurodevelopmental disorders (*N* = 7), adult psychiatric disorders (*N* = 7), neurodegenerative disorders (*N* = 15), epilepsy (*N* = 6), multiple sclerosis (*N* = 4), and other disorders (*N* = 4). An overview of the included studies is given in Supporting Information Table S1 and a flowchart of the systematic selection process can be found in Supporting Information Figure S1.

### Network Size and Imaging Modality Effects

**Figure 2.**
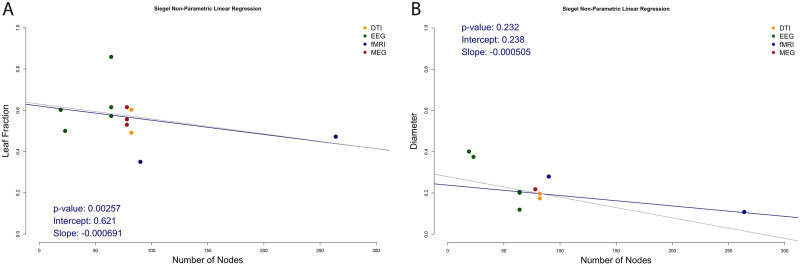
Nonparametric linear regression for number of nodes and normalized leaf fraction (A) and diameter (B). The gray line indicates the same regression, but excluding studies with more than 250 nodes; in this analysis for leaf fraction (2.1) the *p* value is 0.023, with an intercept of 0.63 and a slope of −0.0007. For diameter the *p* value is 0.426, with an intercept of 0.279 and a slope of −0.001.

To analyze network size effects, control groups from studies in adult populations were used. For 12 studies data were available: 5 EEG studies, 2 MEG, 2 fMRI study, 2 DTI study, and 1 combined MEG/MRI study. Regression analysis showed that leaf fraction decreased with the number of nodes in the network (slope = −6.91 × 10^−4^; *p* = 0.00257; Figure 2A). Values for leaf fraction in healthy controls ranged from 0.35 to 0.859. Diameter did not show significant correlation with the number of nodes (slope = −5.05 × 10^−4^; *p* = 0.232; Figure 2B). Values for diameter in healthy controls ranged from 0.108 to 0.401. Insufficient data were available to provide a quantitative analysis of imaging modality effects, frequency band effects in EEG/MEG studies, or effects using different connectivity measures/connection definitions within one modality.

### MST Characteristics of Neurological and Psychiatric Disorders

**Figure 3.**
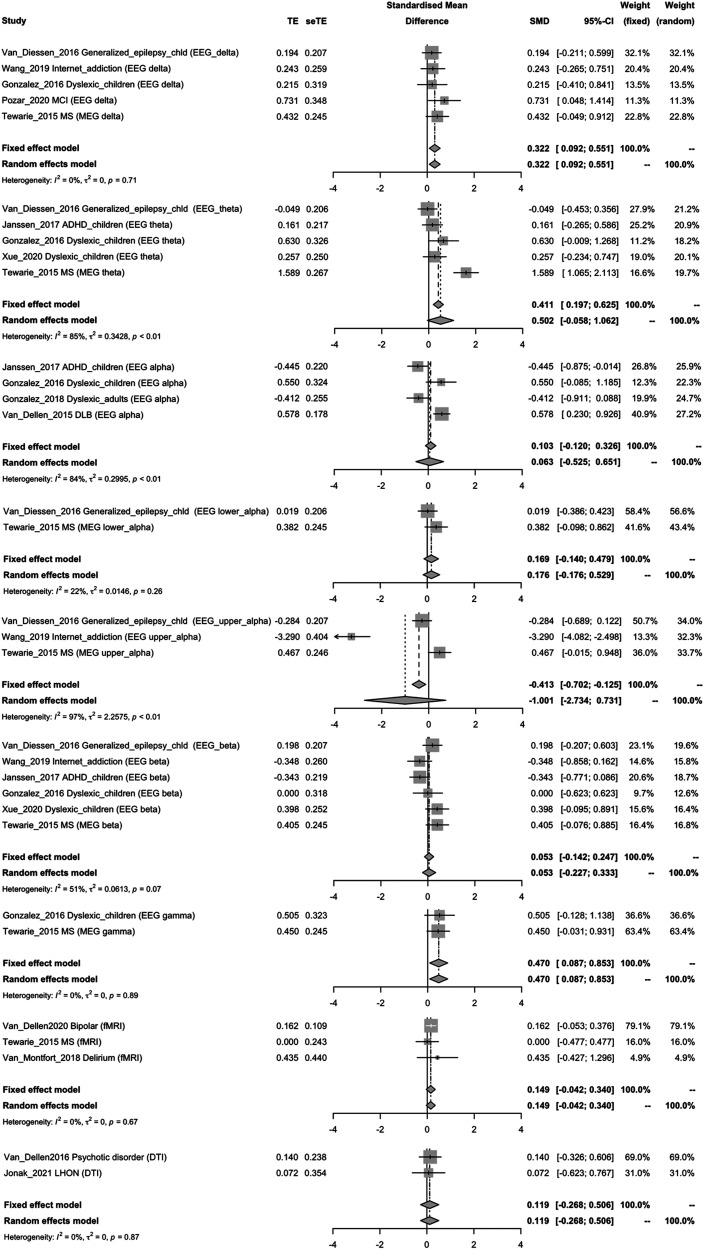
Forest plots for fixed-effect and random-effects meta-analysis on the standardized difference of the mean estimates for mean diameter. Studies are stratified by imaging modality. MEG/EEG studies are aggregated, but stratified for each frequency band. Low heterogeneity indicates that the included studies agree about the magnitude and direction of effect. The *p* value indicates whether the calculated heterogeneity deviates significantly. Meta-analyses with more studies tend to have a higher power to detect significant heterogeneity. SMD, standardized mean difference; 95%-CI, 95% confidence interval; TE, estimate of treatment effect, for example, log hazard ratio or risk difference; seTE, standard error of treatment estimate; ADHD, attention-deficit hyperactivity disorder; LHON, Leber’s hereditary optic neuropathy; MCI, mild cognitive impairment; MS, multiple sclerosis; DLB, dementia with Lewy bodies.

**Figure 4.**
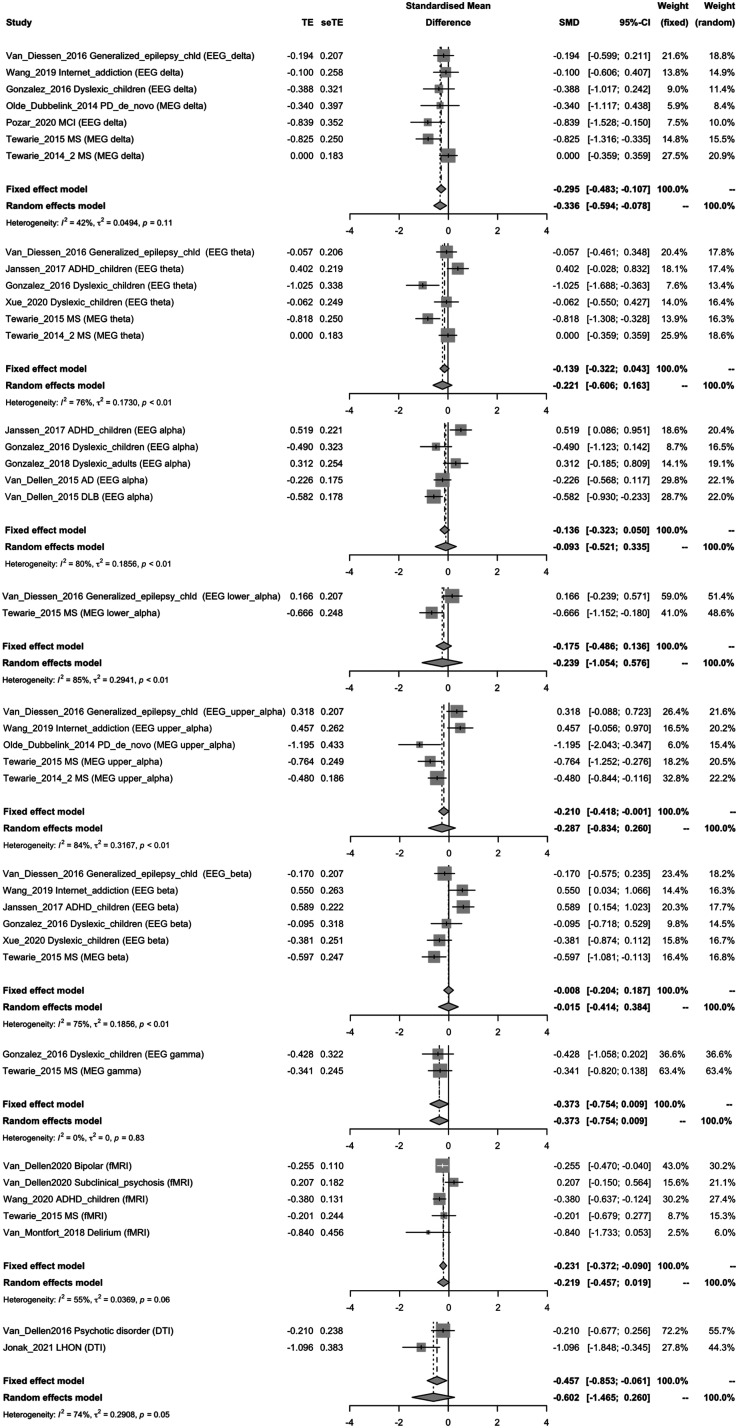
Forest plots for fixed-effect and random-effects meta-analysis on the standardized difference of the mean estimates for mean leaf fraction. Studies are stratified by imaging modality. MEG/EEG studies are aggregated, but stratified for each frequency band. SMD, standardized mean difference; 95%-CI, 95% confidence interval; TE, estimate of treatment effect, for example, log hazard ratio or risk difference; seTE, standard error of treatment estimate; ADHD, attention-deficit hyperactivity disorder; MCI, mild cognitive impairment; AD, Alzheimer’s disease; DLB, dementia with Lewy bodies; LHON, Leber’s hereditary optic neuropathy; MS, multiple sclerosis; PD, Parkinson’s disease.

Data from 16 studies were available to calculate transdiagnostic, standardized effects of brain disorders on MST metrics. Fixed-effect and random-effects meta-analysis were performed on the standardized difference of the mean estimates of mean leaf fraction per study and mean diameter per study. EEG and MEG studies were aggregated per frequency band. fMRI studies and DTI studies were analyzed separately because of unknown imaging modality effects and differences in network size compared to EEG/MEG studies. Figure 3 and Figure 4 show the disease effect for diameter and leaf fraction, respectively.

A significant effect is seen for delta-band diameter (*SMD* = 0.322, 95% CI = 0.092; 0.551) and leaf fraction (fixed effects model: *SMD* = −0.295, 95% CI = −0.483; −0.107; random-effects model *SMD* = −0.336, 95% CI = −0.594; -−0.078), and for diameter in gamma band (*SMD* = 0.470, 95% CI = 0.087; 0.853), implying a cross-disorder effect of a shift toward a more linelike network.

Due to overall levels of heterogeneity (see Figure 3 and Figure 4), we concluded that it is not possible to show any generic disease effects across disorders and modalities on diameter and leaf fraction in our meta-analysis. Because of the small number of studies per disease category, no separate analyses for each disease category was conducted; instead these effects are described in a qualitative matter in the following sections.

### Neurodevelopmental Disorders

We included two studies on attention-deficit/hyperactivity disorder (ADHD), one study on autism spectrum disorder (ASD), three studies on dyslexia, and one study on language-based learning disorder, of which 6 were EEG studies and 1 was an fMRI study. An overview of the mean MST values and standard deviation (*SD*) is included as Supporting Information Table S2. The studies comprised a total number of 261 patients with neurodevelopmental disorders.

Children with ASD showed a lower EEG alpha-band leaf fraction than healthy controls (Zeng et al., 2017). Wang and others found a lower fMRI leaf fraction and kappa and tree hierarchy in children with ADHD compared to normal developing children (Wang et al., 2020). In contrast, Janssen and others found a lower EEG alpha-band diameter and higher leaf fraction and tree hierarchy in children with ADHD than typically developing children and a higher beta-band leaf fraction (Janssen et al., 2017).

Fraga González and others found dyslexic children to have a significantly lower leaf fraction and higher diameter in EEG theta band than typically reading children (Fraga González et al., 2016), while Xue and others found no significant differences (Xue et al., 2020). Fraga González and others found a higher kappa in dyslexic young adults (Fraga González et al., 2018). Infants at risk for developing a language-based learning disorder showed a higher leaf fraction than typically developing children (Zare et al., 2016).

#### Adult psychiatric disorders.

We included seven studies on adult psychiatric disorders, with 483 patients: four studies on psychotic disorders, one study on bipolar disorder and psychotic disorder, one study on major depressive disorder, and one study on internet addiction. Imaging modalities included EEG (*N* = 4), fMRI (*N* = 1), and DTI (*N* = 2). An overview of the available mean MST values and *SD* is included as Supporting Information Table S3.

Anjomshoa and others found a higher DTI diameter and lower kappa and leaf number in schizophrenia patients than healthy controls (Anjomshoa et al., 2016). In contrast, Van Dellen and others found no significant differences in MST topology between patients with a psychotic disorder, individuals with subclinical psychotic symptoms, and healthy controls, neither with fMRI nor with DTI-based networks (van Dellen et al., 2016; van Dellen et al., 2020). Krukow and others reported findings seemingly inconsistent with the studies above, that is, a smaller diameter and leaf fraction in EEG delta and lower gamma band and smaller diameter in EEG beta band. Importantly in this EEG study, all patients were treated with atypical antipsychotics (Krukow et al., 2019). Jonak and others found a higher gamma-band hierarchy but lower beta-band hierarchy in first-episode psychosis patients compared to patients with longer illness duration, but found no differences in diameter, leaf fraction, or kappa between these groups (Jonak et al., 2019).

Bipolar-I patients were found to have a lower fMRI leaf fraction and kappa than controls and patients with subclinical psychosis, and a lower leaf fraction than patients with schizophrenia (van Dellen et al., 2020). This study found no differences in global MST network topology between patients on antipsychotic medication or lithium and those who did not use medication. Li and others found a higher EEG theta-band leaf fraction for patients with major depressive disorder than healthy controls (Li et al., 2017).

Finally, Wang and others found that alpha- and beta-band EEG MST was more starlike in subjects with internet addiction than controls (Wang et al., 2019): a higher kappa and lower diameter correlated with higher addiction severity.

#### Neurodegenerative diseases.

Fifteen studies were included, comprising 760 patients with neurodegenerative disorders: seven EEG, five MEG, two fMRI and one DTI study. An overview of the available mean MST values and *SD* is included as Supporting Information Table S4.

Synucleinopathies are neurodegenerative diseases characterized by the abnormal neural accumulation of alpha-synuclein proteins. Parkinson’s disease (PD) and dementia with Lewy bodies (DLB) are types of synucleinopathies, which may be diagnosed based on the neuroanatomical spreading pattern of the proteins and clinical presentation. Three studies reported MST disturbances in synucleinopathies, which may also reflect disease progression.

Olde Dubbelink and others studied MEG recordings of de novo PD patients, 43 chronic PD patients and 14 controls, and included a follow-up measurement after 4 years (Olde Dubbelink et al., 2014). They found lower leaf fraction and tree hierarchy in the (upper) alpha band in PD patients compared to controls. Leaf number (theta band) and tree hierarchy (delta band) were decreased at follow-up in the PD group. In the control group, alpha-band leaf fraction and beta-band tree hierarchy decreased at follow-up.

Utianski and others also found a lower diameter and higher leaf fraction in EEG delta and theta-band recordings of cognitively normal PD (PD-CN) than healthy controls. Patients with PD and mild cognitive impairment (PD-MCI) or dementia (PDD) had a lower leaf fraction (upper alpha band) when compared to PD-CN patients (Utianski et al., 2016). The (lower alpha band) diameter was also higher in the PDD compared to PD-CN patients.

Peraza and others found higher theta-band diameter and lower alpha-band leaf fraction in EEGs of patients with PDD, DLB, and AD compared to controls, indicating more linelike networks in the patient groups. A classifier between AD and DLB based on MST and connectivity values reached 80% sensitivity and 85% specificity (Peraza et al., 2018). A second study also reported a higher diameter and lower leaf fraction in DLB compared to patients with AD and control subjects, but only in the alpha band (van Dellen et al., 2015).

Of interest, (theta and alpha band) leaf fraction was associated with cognitive decline in cross-sectional analyses of three EEG studies of DLB and PDD patients (Peraza et al., 2018; Utianski et al., 2016; van Dellen et al., 2015). The severity of PD motor symptoms was associated with lower MEG delta-band leaf number and tree hierarchy.

Taken together, these studies found disease effects in different frequency bands. A tendency toward less integrated, more linelike MST topology was reported across studies and was associated with clinical deterioration in synucleinopathies.

AD is the most common cause of dementia, and is subject of six EEG studies that characterize MST topology. Das and Puthankattil found a higher diameter in 13 mildly cognitively impaired AD patients compared to 20 healthy controls across a range of (delta, theta, lower alpha, upper alpha, and beta) frequency bands and in a variety of recording protocols (Das & Puthankattil, 2020). A lower leaf fraction was observed in lower alpha band in AD compared to controls. Yu and others found a similarly higher diameter and lower leaf fraction and kappa in AD patients’ alpha-band EEG recordings compared to subjects with subjective cognitive decline. A lower leaf fraction and kappa in AD compared to patients with the behavioral variant of frontotemporal dementia (bvFTD) (Yu et al., 2016). Studies by van Dellen (EEG alpha band) and Peraza (EEG theta and alpha band) found MST characteristics of AD patients to be in between those of the DLB patients and controls (Peraza et al., 2018; van Dellen et al., 2015).

Five studies reported MST metrics related to at-risk states for AD dementia, with inconsistent results. An fMRI study showed that compared to controls, patients with MCI had a more starlike network, while AD patients had a more linelike network (Wang et al., 2018). Two MEG studies found regional but no global metric differences in MCI patients compared to controls (Jacini et al., 2018; López et al., 2017), while an EEG study found a more linelike MST in the delta band, which contributed to a classifier for MCI versus controls (Požar, Giordani, & Kavcic, 2020).

Sorrentino and others studied MEG networks concerning insulin growth factor-1 (IGF-1), which has been suggested as a brain atrophy marker related to the risk of developing AD (Sorrentino et al., 2017). IGF-1 was correlated with beta-band leaf fraction, tree hierarchy, and theta-band leaf fraction, suggesting an association with less integrated network topology.

Frontotemporal dementia (FTD) is characterized by progressive cell loss in frontal and temporal lobes. One variant of FTD is the behavioral variant mentioned above. Two studies report on MST topology in bvFTD. Yu and others found no MST disturbances in EEG recordings of 48 bvFTD patients compared to subjects with SMC, and, as mentioned above, did find disturbances in AD compared to bvFTD (Yu et al., 2016). In contrast, Saba and others found a higher diameter and lower leaf fraction in resting-state fMRI recordings of patients with bvFTD than controls (Saba et al., 2019). MST findings in FTD thus remain inconclusive.

Fraschini and others compared resting-state EEG networks of 21 patients with amyotrophic lateral sclerosis (ALS) (a degenerative motor neuron disease affecting upper and lower motor neurons) and 16 control patients (Fraschini et al., 2016). A lower beta-band leaf fraction was found in ALS patients compared to controls, and lower leaf fraction, kappa, and tree hierarchy correlated with worse disability scores. In contrast, Sorrentino and others found more pronounced disturbances in MEG recordings of patients with advanced stage ALS (*N* = 24) than early stage ALS (*N* = 26) compared to healthy controls (*N* = 25), with a pattern of higher tree hierarchy, kappa and leaf fraction across frequency bands, suggesting more starlike networks with progressive ALS (Sorrentino et al., 2018).

Finally, Jonak and others found lower leaf fraction and tree hierarchy in DTI networks of 15 Leber’s hereditary optic neuropathy (LHON) patients to 17 controls (Jonak et al., 2021). The more linelike MST in LHON patients correlated with illness duration.

#### Multiple sclerosis.

Tewarie and others performed three subsequent MST studies in multiple sclerosis (MS) using MEG recordings, while Nauta and colleagues performed a fourth analysis partially on the same cohort. An overview of the available mean MST values and *SD* is included as Supporting Information Table S5.

First, Tewarie and others found a frequency-specific effect on MEG networks compared to 21 early MS patients (relapsing-remitting subtype) to 17 controls (Tewarie et al., 2014b). Similarly, diameter was lower in the theta band but higher in the upper alpha band in the MS group, while the opposite pattern was seen for the leaf fraction. Furthermore, the upper alpha-band kappa and hierarchy were lower in the MS group. Taken together, these results pointed toward a more starlike theta-band network but more linelike alpha- and beta-band network in MS patients.

Secondly, this group compared MEG data of 102 MS patients (67% relapsing-remitting subtype, 21% secondary- and 12% primary-progressive subtype) and 42 controls (Tewarie et al., 2014a). Patients showed lower leaf fraction, hierarchy, and kappa than controls in the upper alpha band, but not in other frequency bands. A third study analyzed MEG and fMRI recordings of 86 MS patients (around 6 years after diagnosis) and 21 healthy controls (Tewarie et al., 2015b). They found group differences in MEG recordings but not in fMRI data; MS patients had a lower leaf fraction in the upper alpha band and a lower kappa in the frequencies from 0.5 to 13 Hz. Furthermore, in MS patients, a more linelike topology was associated with clinical disability (delta/theta-band leaf fraction and kappa; lower alpha-band kappa), thalamic atrophy (theta/alpha-band leaf fraction and kappa) and cognitive functioning (alpha-band kappa).

Nauta and others then showed in an extended sample of this cohort that a lower beta-band diameter and lower delta-band leaf fraction predicted 15% of the variance in cognitive decline after 5 years, independent of structural damage. Cross-sectional analyses showed that lower tree hierarchy was especially related to worse cognition, independent of the frequency band (Nauta et al., 2020).

MST studies in MS patients are based on studies from one research group and partially the same cohort of respectable sample size. A complex, frequency-dependent pattern of alterations in MST characteristics emerges from these studies, which relates to clinical impairments, but a straightforward interpretation seems impossible.

#### Epilepsy.

Six studies on MST metrics and epilepsy were included in this review, consisting of four EEG studies, one MEG study, and one fMRI study, with a total number of 231 patients. An overview of the available mean MST values and *SD* is included as Supporting Information Table S6. All studies focused on interictal recordings. Three studies included patients with childhood epilepsy.

Van Diessen and others found a higher MST diameter and lower leaf fraction in the delta band in EEGs of drug-naïve children with newly diagnosed focal epilepsy than in controls (van Diessen et al., 2016). They found an opposite difference in topology in the upper alpha band, with a lower diameter and higher leaf fraction in the focal epilepsy group. No differences were found when comparing children with generalized epilepsy to the focal epilepsy group or controls.

The same authors studied the effect of sleep deprivation, which lowers the seizure threshold and increases interictal EEG abnormalities, on functional EEG networks in children with focal epilepsy compared to age-matched controls (van Diessen et al., 2014). Alpha-band diameter increased and the leaf fraction decreased after sleep deprivation in patients, while the opposite pattern was seen in controls. They speculated that this shift in network organization after sleep deprivation in epilepsy patients follows literature showing a more regular alpha-band network organization during the ictal state; sleep deprivation may thus cause a shift toward an ictal network state. Kinney-Lang and others analyzed the EEG networks of preschool children with epilepsy (both focal and generalized) and cognitive impairment (Kinney-Lang et al., 2019). They found that worse performance on cognitive tasks was associated with lower (alpha/beta band) diameter and higher leaf fraction.

Interictal MST characteristics may be associated with disease severity and treatment resistance. DeSalvo and others studied fMRI data of 40 patients with medically intractable temporal lobe epilepsy who were to undergo epilepsy surgery. They found that preoperative MST topology differed between patients who became seizure-free after surgery as compared to patients who would not (DeSalvo et al., 2020). Leaf fraction was 9% lower and tree hierarchy was 10% lower in patients with ongoing seizures than in seizure-free patients, suggesting less integrated networks in patients with worse outcomes. A similar finding was reported by Van Dellen and colleagues, where the preoperative diameter in MEG recordings of patients with lesional epilepsy correlated with higher seizure frequency (4–10 Hz) (van Dellen et al., 2013). The alpha-band leaf fraction increased in patients after successful epilepsy surgery, but remained unchanged after surgery in patients with ongoing seizures. A third study reported on adults with pharmacoresistant epilepsy and found that theta-band MST diameter decreased in responders to vagal nerve stimulation as an add-on treatment, but not in nonresponders (Fraschini et al., 2014). These findings indicate that successful epilepsy treatment is associated with a recovery toward a more integrated (i.e., more starlike) network organization.

#### Other studies.

We included four studies with a total of 98 patients with other disorders, that is, delirium (*N* = 2), migraine (*N* = 1) and meningioma (*N* = 1), including one EEG, two MEG, and one fMRI study. An overview of the available mean MST values and *SD* is included as Supporting Information Table S7.

One EEG and one fMRI study analyzed the MST topology related to delirium. Numan and others studied EEG recordings of patients who had undergone cardiac surgery and compared patients who developed hypoactive delirium to patients without delirium (Numan et al., 2017). They found a lower alpha-band leaf fraction in patients with hypoactive delirium compared to the nondelirious controls. A resting-state fMRI study by Van Montfort and others similarly found a higher diameter and lower leaf fraction in brain networks of patients during delirium, which normalized after delirium resolved (van Montfort et al., 2018). In this pilot study of nine patients, a lower leaf fraction and tree hierarchy correlated to a longer delirium duration, used as a proxy for syndrome severity. Both MST studies thus indicate a less integrated network during a state of delirium.

One MEG study compared patients with migraine to controls but found no group differences in global MST metrics (Nieboer et al., 2020). Finally, Van Nieuwenhuizen and others found a lower theta-band maximum MST degree, but no other differences in global MST metrics, in MEG recordings of 20 meningioma patients compared to healthy controls (van Nieuwenhuizen et al., 2018).

## DISCUSSION

This systematic review shows that the past decade of MST analysis has not yet led to definitive neuropsychiatric symptoms or disease biomarkers. This finding may partially explain network size and modality differences across studies: MST leaf fraction (but not diameter) was found to correlate with network size significantly.

Previous meta-analyses on cross-disorder data have shown general disease effects on brain networks across different neuropsychiatric disorders (Crossley et al., 2014). For Alzheimer’s disease and epilepsy, reviews have suggested modality-invariant disease effects on network characteristics (Tijms et al., 2013; Van Diessen et al., 2013). We therefore assessed whether MST characteristics show modality- and disorder-independent alterations in neuropsychiatric disorders. Our meta-analyses suggest a cross-disorder shift toward a more linelike topology in EEG/MEG delta and gamma band. We found no significant heterogeneity for these frequencies. Still, due to the relatively small number of included studies that had small sample sizes, heterogeneity analysis may not have been sufficiently powered.

Insufficient data were available for stratified analysis based on imaging modality, node and connection definition, or study population. Nevertheless, this review and meta-analysis has several implications for the methodological approach in network neuroscience and a transdiagnostic perspective on network alterations in brain disease. Since a straightforward, transdiagnostic interpretation was not possible for all disorder categories, only the disorders that suggested a trend are discussed below.

### Network Size and Modality Effects

The effects of network size and other differences in methodology most likely hamper reproducibility necessary for the development of biomarkers in the field of network neuroscience. The comparison of MST metrics in control populations across studies showed that the MST leaf fraction but not the diameter decreases with increasing network size. Furthermore, the range of values for both measures was large (leaf fraction = 0.35–0.859 and diameter = 0.108–0.401), further illustrating effects of methodological differences across studies. MST analysis was most frequently applied to band-pass filtered neurophysiological recordings, but applications to functional and structural MRI scans have also been reported. Some individual studies did show substantially different metric values, suggesting that as expected, the specific processing pipeline before MST reconstruction impacts the estimated MST topology. In addition, for EEG and MEG data, it is important which connectivity measure is used. For example, phase-based measures such as the phase-lag index might be noisier than amplitude-based measures such as the amplitude envelop correlation, which could reduce the ability to extract consistent functional connectivity and subsequently influence MST parameters (Colclough et al., 2016). Lastly, harmonization of node definitions may help to increase comparability between studies and across modalities (Tewarie et al., 2015a).

### Neurodevelopment and Neurodegeneration

During maturation, MST topology shifts from a linelike, decentralized topology toward a more starlike (integrated) topology, while the inverted age-relation is seen with aging in the fifth-sixth decade (Boersma et al., 2013; Otte et al., 2015; Smit et al., 2016; van Dellen et al., 2018). We found no systematic pattern of network disturbances in the scarce MST literature on neurodevelopmental disorders. Studies on neurodegeneration suggest a shift toward a more linelike MST topology as a general characteristic in neurophysiological recordings, which is in line with broader findings in brain-aging literature (Fjell et al., 2014). MST characteristics in neuroimaging modalities remain understudied. Studies in ALS and patients at risk for (AD) dementia show inconsistent findings; the latter outcome of this review suggests that currently used MST characteristics are not a promising predictive biomarker in at-risk groups for developing dementia.

Interestingly, different subtypes of neurodegenerative disease seem to vary in damage to MST organization. A pattern emerges of MST topology from starlike to linelike with HC < bvFTD < AD < DLB < PDD. MST disturbances in neurophysiological recordings may thus be a marker of disease progression and symptom severity in synucleinopathies.

### Epilepsy and Attention Disorders

Preliminary evidence showing a loss of functional network integration in frequencies below 10 Hz is reported in association with focal epilepsy, which may be further provoked by sleep deprivation. Interestingly, successful treatment seems to be associated with increased network integration in this frequency range. Increased network integration in the upper alpha band is found in focal childhood epilepsy. Increased network integration in frequencies above 10 Hz may be associated with cognitive impairment in patients with childhood epilepsy.

In disorders characterized by disturbances in the cognitive domain of attention, a loss of alpha-band network integration (lower leaf fraction, higher diameter) emerges as a recurrent finding. It has been reported in delirium, in one study in ADHD (although the opposite finding was also reported in ADHD), and DLB. Future studies may reveal if interventions that increase alpha-band MST network integration and efficiency may be used to treat attention deficits, similar to early findings in epilepsy patients showing a network normalization after seizure freedom. Of interest, van Lutterveld and others, found a higher MST maximum centrality in the EEG alpha band in experienced meditators than in novice meditators, and leaf fraction tended to be higher in this group (van Lutterveld et al., 2017). Applications of this and other interventions in clinical populations are needed to test if sufficient alpha-band network integration is a prerequisite for attentional tasks, and if interventions aiming to improve these characteristics specifically can be used in clinical care.

### Limitations

Our approach to reviewing and interpreting MST studies’ findings across modalities has several limitations. First, several studies did not report actual MST metric values or effect sizes, limiting the interpretability of findings. The limited availability of quantitative data makes it premature to conclude if there is no pattern of consistent network disturbances across disorders, or if data on this topic is simply underpowered. Secondly, most studies solely mentioned significant values, leading to a positive outcome bias. We suggest that reporting guidelines are needed in network neuroscience that emphasize reporting the numeric values for network metrics as completely as possible.

We did not include node-specific MST metrics in this review; we found no indication that consistent findings were reported with this approach, but the macroscale MST metrics analysis may only be less sensitive to disease-specific effects on brain networks. Other graph theoretical approaches bring complementary clinical insights, including individual nodal, edge and modular characteristics.

Finally, we aimed to gain transdiagnostic insights from different studies with variable methodology in the definition of nodes and edges, imaging modalities, and frequency bands in MEG/EEG recordings; possible confounds due to (differences in) processing pipelines are no longer apparent in our modality-invariant summation of these studies.

We used a transdiagnostic approach to neuropsychiatric pathology. There is increasing interest in overarching mechanisms that are a final common pathway to general factors of psychopathology. These include the *p* factor for psychopathology, the characterization of psychiatric disorders from a symptom-network perspective, and studies of general cognitive dysfunction based on graph analysis (Borsboom, 2017; Caspi et al., 2014; Crossley et al., 2014; Menon, 2020; van Bork et al., 2017). Another, complementary approach that may advance the field is to look for convergence of evidence in isolating disease-specific effects by comparing different network analysis approaches. Such within-disease approaches may for example contribute to the development of staging or subtyping in specific pathological conditions, and may help facilitate precision medicine approaches.

### Conclusion

The MST approach has proven fruitful in capturing disease-related changes in brain network topology. Harmonization of node definitions and especially network size remains a prerequisite for comparing findings across studies and modalities. Empirical findings are more consistent in neurological (in particular neurodegenerative) than psychiatric disorders and neurodevelopmental disorders. They show that alterations in network topology are found across disorders even after strict correction for network density effects. Most consistent (but still preliminary) evidence was found for MST measures as markers of attention disorders, particularly in epilepsy, and as markers of disease progression in neurodegenerative disease. Importantly, contradicting findings within clinical populations were shown in previous reviews on conventional graph analysis, for example, in Alzheimer’s disease and epilepsy; such contradictions were not found in the MST literature to date (Tijms et al., 2013; Van Diessen et al., 2013). There is currently insufficient evidence for the use of MST metrics as sensitive and specific biomarkers for neuropsychiatric disorders.

## SUPPORTING INFORMATION

Supporting information for this article is available at https://doi.org/10.1162/netn_a_00245.

## AUTHOR CONTRIBUTIONS

Edwin van Dellen: Conceptualization; Data curation; Investigation; Methodology; Supervision; Visualization; Writing – original draft; Writing – review & editing. Bart de Rooy: Conceptualization; Data curation; Methodology; Project administration; Resources; Visualization; Writing – original draft; Writing – review & editing. Nicky Blomsma: Conceptualization; Data curation; Formal analysis; Investigation; Methodology; Project administration; Visualization; Writing – original draft; Writing – review & editing. Rick van der Spek: Conceptualization; Formal analysis; Methodology; Software; Visualization; Writing – review & editing. Frank Gerritse: Conceptualization; Data curation; Project administration; Writing – original draft. Prejaas Tewarie: Conceptualization; Methodology; Writing – review & editing. Arjan Hillebrand: Conceptualization; Methodology; Writing – review & editing. Wim Otte: Conceptualization; Methodology; Writing – review & editing. Cornelis Jan Stam: Conceptualization; Methodology; Writing – review & editing.

## FUNDING INFORMATION

Edwin van Dellen, ZonMw (https://dx.doi.org/10.13039/501100001826), Award ID: 60-63600-98-711. Edwin van Dellen, UMC Utrecht Clinical Research Talent Fellowship.

## TECHNICAL TERMS

Minimum spanning tree: A spanning tree is defined as a subgraph that includes all *N* nodes of the original graph and *N* − 1 links (m). When the sum of the weights of the links is minimized, this is called a minimum spanning tree of the connected weighted graph.
Diameter: The largest distance (in number of edges) between any two nodes, normalized for the total number of edges. Diameter is a measure of network efficiency. An increase in diameter, means a decrease in global efficiency, whereas a low diameter indicates a more efficient information flow between brain regions.
Leaf fraction: The leaf fraction (L_f_) is the leaf number (L) divided by the maximum possible leaf number. Leaf fraction is considered measure of centrality.
Leaf number: The number of nodes in graph with only one connection.
Kappa: A measure that relates to the spread of information across the tree. When kappa is low there is a low number of highly connected nodes.
Tree hierarchy: Tree hierarchy quantifies the trade-off between large-scale integration in the minimum spanning tree and the overload of central nodes.
