# Supplementary material for: Minimum spanning tree analysis of brain networks: A systematic review of network size effects, sensitivity for neuropsychiatric pathology, and disorder specificity
Source: Netw Neurosci. 2022 Jun 1;6(2):301–19. doi: 10.1162/netn_a_00245 (PMC9207994; doi:10.1162/netn_a_00245)
Supplement: Supplementary file 1 [file netn-06-301-s001.pdf]

Blomsma, N., de Rooy, B., Gerritse, F., van der Spek, R., Tewarie, P., Hillebrand, A., Otte, W. M., Stam, C. J. & van Dellen, E. (2022). Supporting information for "Minimum spanning tree analysis of brain networks: a systematic review of network size effects, sensitivity for neuropsychiatric pathology and disorder specificity." *Network Neuroscience.* Advance publication. https://doi.org/10.1162/netn_a_00245

Search terms

Pubmed search terms:

((connectivity[Title/Abstract] OR network[Title/Abstract] OR networks[Title/Abstract] OR subnetwork[Title/Abstract] OR subnetworks[Title/Abstract] OR graph[Title/Abstract] OR graphs[Title/Abstract] OR graph OR disconnection[Title/Abstract] OR dysconnection[Title/Abstract] OR connectome[Mesh] OR connectome[Title/Abstract] OR network[Title/Abstract] OR networks[Title/Abstract] OR subnetwork[Title/Abstract] OR subnetworks[Title/Abstract] OR dysconnection[Title/Abstract] OR connectomics[Title/Abstract] OR connectomic[Title/Abstract] OR neural communication[Title/Abstract] OR FC)) **AND** (spanning tree*[Title/Abstract] OR spanning graph*[Title/Abstract] OR MST[Title/Abstract] OR MWST)

Embase search terms:

(connectivity:ab,ti OR graph:ab,ti OR graphs:ab,ti OR disconnection:ab,ti OR connectome:ab,ti OR connectomes:ab,ti OR network:ab,ti OR networks:ab,ti OR subnetwork:ab,ti OR subnetworks:ab,ti OR connectomic:ab,ti OR connectomics:ab,ti OR 'neural communication':ab,ti OR fc:ab,ti) **AND** ('spanning tree':ab,ti OR 'spanning graph':ab,ti OR mst:ab,ti OR mwst:ab,ti)

Supplementary table 1: Overview of included studies

Category	*First Author*	*Clinical group*	*N*	*Modality*	*Nodes*
**Neurodevelopmental disorders**	*Janssen 2017*	Children with ADHD<br>Typically developing children	42<br>43	EEG	78
	Wang 2020	Children with ADHD<br>Typically developing children	102<br>143	fMRI	264
	González 2016	Dyslexic children<br>Typically reading children	29<br>15	EEG	64
	González 2018	Dyslexic adults<br>Typical readers	28<br>36	EEG	64
	Xue 2020	Dyslexic children<br>Typically reading children	27<br>40	EEG	30
	Zare 2016	Infants with a family history of language learning disorder<br>Typically developing infants	12<br>12	EEG	62
	Zeng 2017	High-functioning autism children<br>Typically developing children	21<br>21	EEG	128
**Adult psychiatric disorders**	*Krukow 2019*	Schizophrenia patients<br>Healthy control	35<br>35	EEG	19
	Jonak 2019	First episode psychosis<br>Long duration illnes psychosis	30<br>30	EEG	21
	Van Dellen 2020	Bipolar-I disorder<br>Schizophrenia spectrum patients<br>Sub-clinical psychosis<br>Healthy control	136<br>97<br>35<br>219	fMRI	264
	Anjomshoa 2016	Schizophrenia patients<br>Healthy control	27<br>19	DTI	116
	Van Dellen 2016	Sub-clinical psychosis<br>Psychotic disorder<br>Healthy control	35<br>35<br>36	DTI	82

	Li<br>*2017*	Major depressive disorder<br>Healthy control	23<br>15	EEG	72
	Wang<br>2019	Internet addiction<br>Healthy control	30<br>30	EEG	64
**Neurodegenerative diseases**	*Van Dellen*<br>*2015*	Lewie body dementia<br>Alzheimers Disease<br>Control	66<br>66<br>66	EEG	19
	Olde Dubbelink<br>*2014*	Parkinsons disease<br>*Parkinsons disease de novo (subgroup)*<br>Controls	43<br>*12*<br>14	MEG	78
	Yu<br>*2016*	Alzheimers<br>Frontotemporal dementia<br>Subjective cognitive decline	69<br>48<br>64	EEG	21
	Das<br>*2020*	Mild cognitive impaired Alzheimers<br>Healthy controls	13<br>20	EEG	23
	Utianski<br>*2016*	Parkinsons disease cognitively normal<br>Parkinsons disease MCI<br>Parkinsons disease dementia<br>Healthy control	57<br>13<br>18<br>57	EEG	21
	Saba<br>*2019*	Frontotemporal Dementia<br>Healthy control	41<br>39	fMRI	116
	Peraza<br>*2018*	Alzheimers disease<br>Dementia with Lewy bodies<br>Parkinson's disease dementia<br>Healthy controls	16<br>25<br>21<br>17	EEG	128
	Pozar<br>*2020*	Mild cognitive impairment<br>Cognitively normal	13<br>27	EEG	64
	Fraschini<br>*2016*	Amyotrophic lateral sclerosis<br>Healthy controls	21<br>16	EEG	58
	Sorrentino<br>*2018*	Advanced amyotrophic lateral sclerosis<br>Early amyotrophic lateral sclerosis	24<br>26	MEG	90

		Healthy controls	25		
	Sorrentino 2017	Subjects from AGHLS	156	MEG	80
	Jacini 2018	Amnestic mild cognitive impairment<br>Healthy control	16<br>16	MEG	90
	Lopez 2017	Mild cognitive impairment<br>Healthy control	29<br>29	MEG	78
	Wang 2018	Alzheimer's disease<br>Mild cognitive impairment<br>Healthy control	27<br>62<br>29	fMRI	90
	Jonak 2021	LHON<br>Healthy controls	15<br>17	DTI	82
**Multiple sclerosis**	*Tewarie 2014 (1)*	Multiple sclerosis<br>Healthy control	21<br>17	MEG	78
	Tewarie 2015	Multiple sclerosis<br>Healthy control	86<br>21	MEG/fMRI	78
	Tewarie 2014 (2)	Multiple sclerosis<br>Healthy control	102<br>42	MEG	78
	Nauta 2020	Multiple sclerosis/ follow-up	146<br>/100	MEG	78
**Epilepsy**	*Fraschini 2014*	Pharmaco resistant epilepsy<br>Nonresponder to VNS<br>Responder to VNS	 5<br>5	EEG	19
	Van Diessen 2016	Drug naive chld generalized epilepsy<br>Drug naive chld focal epilepsy<br>Healthy control	27<br>62<br>179	EEG	17
	Van Dellen 2014	Meningioma with seizures<br>*Seizure free post surgery*<br>*Seizures post surgery*	 13<br>7	MEG	78
	Van Diessen 2014	chld. suspected new onset focal epilepsy<br>Control	21<br>17	EEG	21

		sleep deprevation (design)			
	Kinney-Lang 2019	Children with early onset epilepsy<br>Cognitively normal<br>Mild cognitive impairment<br>Severe cognitive impairment	 31<br>7<br>13	EEG	20
	DeSalvo 2020	Temporal lobe epilepsy (TLE)<br>TLE pre-op, seizures post-op<br>TLE pre-op, seizure free post-op	40	fMRI	83
**Other**	*Van Montfort 2018*	Delirium<br>Post Delirium<br>Non delirious control	9<br>7<br>13	fMRI	90
	Numan 2017	Hypo-active delirium<br>Recovering from anesthesia<br>Non delirious control	18<br>20<br>20	EEG	17
	Nieboer 2020	Migraine<br>*Recently diagnosed (subgroup)*<br>*Subgroup long-term illnes (subgroup)*<br>Healthy control	24<br>*12*<br>*12*<br>24	MEG	78
	Van Nieuwenhuizen 2018	Meningioma patients<br>Healthy control	20<br>20	MEG	78

Included articles are arranged by disease category. Nodes: the number of nodes in the network.

ADHD: attention deficit hyperactivity disorder, LLD: language learning disorder, MCI: mild cognitive impairment, AGHLS: Amsterdam Growth and Health Longitudinal Study, LHON: Leber’s hereditary optic neuropathy, VNS: vagal nerve stimulation

Supplementary table 2: MST metrics from studies on Neurodevelopmental disorders

**Modality**	***First Author***	**Clinical group**	**Diameter**	**LF**	**Kappa**	**Th**
EEG delta	*González 2016*	Dyslexic children	0.219 ± 0.014	0.576 ± 0.020	3.501 ± 0.270	0.417 ± 0.023
		TRC	0.216 ± 0.013	0.583 ± 0.012	3.551 ± 0.259	0.418 ± 0.015
	Zare 2016	Infants with a family history of LLD	N/A	N/A[a b]	N/A	N/A[a b]
		TDI	N/A	N/A [a b]	N/A	
EEG theta	*González 2016*	Dyslexic children	**0.224 ± 0.011**	**0.569 ± 0.015**	3.341 ± 0.165	0.412 ± 0.016
		TRC	**0.216 ± 0.015**	**0.584 ± 0.013**	3.415 ± 0.149	0.419 ± 0.015
	Janssen 2017	ADHD children	0.234 ± 0.018	0.491 ± 0.025	N/A	0.370 ± 0.020
		TDC	0.237 ± 0.018	0.482 ± 0.019	N/A	0.364 ± 0.019
	Xue 2020	Dyslexic children	0.360 ± 0.016	0.544 ± 0.016	2.920 ± 0.109	0.392 ± 0.010
		TRC	0.356 ± 0.015	0.545 ± 0.016	2.945 ± 0.099	0.390 ± 0.012
	Wang 2019	Internet addicition	**0.064 ± 0.009**	**0.957 ± 0.086**	**26.467 ± 8.301**	0.498 ± 0.025
		Control	**0.094 ± 0.009**	**0.919 ± 0.078**	**21.402 ± 8.743**	0.498 ± 0.026
EEG alpha	*González 2016*	Dyslexic children	0.206 ± 0.015	0.609 ± 0.027	3.892 ± 0.466	0.432 ± 0.024
		TRC	0.197 ± 0.018	0.623 ± 0.030	3.967 ± 0.398	0.441 ± 0.018
	Janssen 2017	ADHD children	**0.220 ± 0.018**	**0.508 ± 0.023**	N/A	**0.379 ± 0.018**
		TDC	**0.230 ± 0.023**	**0.494 ± 0.030**	N/A	**0.369 ± 0.023**
	Zeng 2017	High-functioning autism children	N/A	N/A[a]	N/A	N/S
		TDC	N/A	N/A [a]	N/A	N/S
	González 2018	Dyslexic adults	0.201 ± 0.012	0.623 ± 0.027	**4.061 ± 0.443**	0.437 ± 0.015
		Typical readers	0.206 ± 0.012	0.615 ± 0.024	**3.857 ± 0.289**	0.437 ± 0.015
EEG beta	*González 2016*	Dyslexic children	0.216 ± 0.012	0.580 ± 0.022	3.542 ± 0.275	0.417 ± 0.020
		TRC	0.216 ± 0.013	0.582 ± 0.018	3.516 ± 0.244	0.425 ± 0.015
	Janssen 2017	ADHD children	0.220 ± 0.023	**0.502 ± 0.030**	N/A	0.371 ± 0.023

		TDC	0.227 ± 0.019	**0.485 ± 0.027**	N/A	0.363 ± 0.019
	Xue 2020	Dyslexic children	0.359 ± 0.013	0.531 ± 0.015	2.863 ± 0.098	0.384 ± 0.012
		TRC	0.354 ± 0.012	0.538 ± 0.020	2.907 ± 0.125	0.383 ± 0.014
EEG gamma	*González 2016*	Dyslexic children	0.205 ± 0.019	0.621 ± 0.038	4.468 ± 1.381	0.431 ± 0.020
		TRC	0.196 ± 0.014	0.637 ± 0.034	4.587 ± 0.875	0.443 ± 0.020
fMRI	*Wang 2020*	ADHD children	N/A	**0.421 ± 0.023**	**1.670 ± 0.099**	**1.304 ± 0.103**
		TDC	N/A	**0.430 ± 0.024**	**1.706 ± 0.098**	**1.344 ± 0.115**

Data are presented as mean and when available SD. (SE) indicates the use of standard error instead of SD.

Bold text represents significant results ($p < 0.05$). For EEG only frequency bands for which data was available are displayed.

N/A: not available, measure not described in study; N/A [a] : significant difference for pathology compared to control group is described in study, but numeric data not available; N/S: not significant; [b] :comparison based on the leaf number, significant compared to controls,

EEG: electroencephalography, fMRI: functional magnetic resonance imaging, ADHD: Attention-deficit/hyperactivity disorder, TDC: typically developing children, TRC: typically reading children, TDI: typically developing infants, LLD: language-learning disorder.

Supplementary table 3: MST metrics from studies on adult psychiatric disorders

Modality	***First Author***	Clinical group	Diameter	LF	Kappa	Th
EEG delta	*Krukow 2019*	SCZ	N/A[a]	N/A[a]	N/A	N/A
		Control	N/A	N/A	N/A	N/A
	Jonak 2019	FES	0.416	0.564	N/A	0.358
		LDI	0.412	0.539	N/A	0.338
	Wang 2019	Internet addicition	0.131 ± 0.054	0.849 ± 0.100	12.478 ± 6.730	0.513 ± 0.058
		Control	0.119 ± 0.043	0.859 ± 0.098	12.043 ± 6.179	0.527 ± 0.071
EEG theta	*Li 2017*	Major depressive disorder	N/A	**0.598**	N/A	N/A
		Control	N/A	**0.544**	N/A	N/A
	Krukow 2019	SCZ	N/S	N/S	N/A	N/A
		Control	N/S	N/S	N/A	N/A
	Jonak 2019	FES	0.436	0.524	N/A	0.332
		LDI	0.396	0.531	N/A	0.324
	Wang 2019	Internet addicition	**0.064 ± 0.009**	**0.957 ± 0.086**	**26.467 ± 8.301**	0.498 ± 0.025
		Control	**0.094 ± 0.009**	**0.919 ± 0.078**	**21.402 ± 8.743**	0.498 ± 0.026
EEG lower alpha	*Krukow 2019*	SCZ	N/S	N/S	N/A	N/A
		Control	N/S	N/S	N/A	N/A
	Jonak 2019	FES	0.446	0,513	N/A	0.363
		LDI	0.420	0.563	N/A	0.353
EEG upper alpha	*Krukow 2019*	SCZ	N/S	N/S	N/A	N/A
		Control	N/S	N/S	N/A	N/A
	Jonak 2019	FES	0.432	0.516	N/A	0.327
		LDI	0.398	0.556	N/A	0.347
	Wang 2019	Internet addicition	**0.064 ± 0.009**	**0.957 ± 0.086**	**26.467 ± 8.301**	0.498 ± 0.025
		Control	**0.094 ± 0.009**	**0.919 ± 0.078**	**21.402 ± 8.743**	0.498 ± 0.026
EEG beta	*Krukow 2019*	SCZ	N/A[a]	N/S	N/A	N/A
		Control	N/A[a]	N/S	N/A	N/A

	Jonak 2019	FES	0.445	0.533	N/A	**0.330**
		LDI	0.399	0.606	N/A	**0.408**
	Wang 2019	Internet addicition	**0.066 ± 0.034**	0.961 ± 0.041	**25.014 ± 7.271**	0.509 ± 0.044
		Control	**0.090 ± 0.053**	0.928 ± 0.073	**19.480 ± 8.141**	0.520 ± 0.063
EEG gamma	*Krukow 2019*	SCZ	N/A[a]	N/A[a]	N/A	N/A
		Control	N/A[a]	N/A[a]	N/A	N/A
	Jonak 2019	FES	0.413	0.548	N/A	**0.373**
		LDI	0.399	0.553	N/A	**0.277**
DTI	*Anjomshoa 2016*	SCZ	**0.566***	**0.451***	**2.779**	N/A
		Control	**0.525***	**0.492***	**2.869**	N/A
	Van Dellen 2016	Sub-clinical psychosis	0.192 ± 0.029	0.492 ± 0.028	2.932 ± 0.180	0.361 ± 0.033
		Psychotic disorder	0.201 ± 0.026	0.483 ± 0.034	2.938 ± 0.214	0.359 ± 0.041
		Control	0.197 ± 0.029	0.491 ± 0.041	2.905 ± 0.163	0.3627 ± 0.037
fMRI	*Van Dellen 2020*	Bipolar-I disorder	0.111 ± 0.002 (SE)	**0.465 ± 0.002 (SE)**	**2.802 ± 0.002 (SE)**	N/A
		Schizophrenia spectrum patients	0.111 ± 0.002 (SE)	**0.471 ± 0.003 (SE)**	**2.835 ± 0.004 (SE)**	N/A
		Sub-clinical psychosis	0.108 ± 0.003 (SE)	**0.478 ± 0.004 (SE)**	**2.867 ± 0.003 (SE)**	N/A
		Control	0.108 ± 0.001 (SE)	**0.472 ± 0.002 (SE)**	**2.840 ± 0.008 (SE)**	N/A

Data are presented as mean and when available SD. (SE) indicates the use of standard error instead of SD. Bold text represents significant results ($p < 0.05$). N/A: not available: measure not described in study, N/A[a] significant difference for pathology compared to control group is described in study, but numeric data not available; N/S: not significant; *normalized values calculated from the raw values of the article.

EEG: electroencephalography, fMRI: functional magnetic resonance imaging, DTI: diffusion tensor imaging.

Supplementary table 4: MST metrics from studies on neurodegenerative disorders

Modality	*First Author*	Clinical group	Diameter	LF	Kappa	Th
EEG delta	*Das 2020*	EC MCI-AD	**0.475 ± 0.079**	0.42 ±0.026	N/A	N/S
		EC control	**0.375 ± 0.079**	0.5 ± 0.158	N/A	N/S
		EO MCI-AD	**0.46 ± 0.078**	0.43 ± 0.026	N/A	N/S
		EO control	**0.37 ± 0.095**	0.505 ± 0.174	N/A	N/S
		MAEC MCI-AD	**0.515 ±0.058**	0.440 ± 0.084	N/A	N/S
		MAEC control	**0.375 ±0.079**	0.500 ± 0.158	N/A	N/S
		MAEO MCI-AD	**0.515 ± 0.058**	0.415 ± 0.075	N/A	N/S
		MAEO control	**0.37 ± 0.095**	0.505 ±0.174	N/A	N/S
	Utianski 2016	PD-CN	**0.42**	**0.56**	N/A	N/A
		PD-D	0.41	0.57	N/A	N/A
		PD-MCI	0.42	0.56	N/A	N/A
		Healthy control	**0.44**	**0.54**	N/A	N/A
	Pozar 2020	MCI	**0.2344 ± 0.0326**	**0.5324 ± 0.0314**	**3.0417 ± 0.2523**	0.3895 ± 0.0471
		Healthy control	**0.2010 ± 0.0494**	**0.5720 ± 0.0517**	**3.6837 ± 0.9523**	0.3937 ± 0.0458
EEG theta	*Das 2020*	EC MCI-AD	**0.505 ± 0.093**	0.395 ± 0.072	N/A	N/S
		EC control	**0.375 ±0.079**	0.500 ± 0.158	N/A	N/S
		EO MCI-AD	**0.56 ±0.097**	0.420 ± 0.072	N/A	N/S
		EO control	**0.37 ±0.095**	0.505 ± 0.072	N/A	N/S
		MAEC MCI-AD	**0.505 ±0.072**	0.490 ± 0.184	N/A	N/S
		MAEC control	**0.375 ±0.079**	0.500 ± 0.072	N/A	N/S
		MAEO MCI-AD	**0.525 ±0.058**	0.440 ± 0.077	N/A	N/S
		MAEO control	**0.37 ±0.095**	0.505 ±0.174	N/A	N/S
	Utianski 2016	PD-CN	**0.40**	**0.58**	N/A	N/A
		PD-D	0.40	0.57	N/A	N/A
		PD-MCI	0.41	0.57	N/A	N/A

		Healthy control	**0.41**	**0.568**	N/A	N/A
EEG alpha	*Van Dellen 2015*	DLB	**0.429 ± 0.044**	**0.568 ± 0.049**	N/A	0.392 ± 0.029
		AD	0.412 ± 0.046	0.588 ± 0.057	N/A	0.399 ± 0.034
		Healthy control	0.401 ± 0.052	0.602 ± 0.066	N/A	0.401 ± 0.037
	Yu 2016	AD	**0.3933**	**0.5838**	**2.996**	0.4035
		FTD	0.3823	0.6062	3.222	0.4077
		SCD	0.3769	0.6069	3.159	0.4098
EEG lower alpha	*Das 2020*	EC MCI-AD	**0.477 ±0.077**	0.530 ± 0.223	N/A	N/S
		EC control	**0.375 ±0.079**	0.500 ±0.158	N/A	N/S
		EO MCI-AD	**0.5 2± 0.248**	**0.311 ± 0.041**	N/A	N/S
		EO control	**0.37 ±0.159**	**0.505 ± 0.174**	N/A	N/S
		MAEC MCI-AD	**0.49 ±0.074**	0.450 ±0.078	N/A	N/S
		MAEC control	**0.375 ±0.079**	0.500 ±0.158	N/A	N/S
		MAEO MCI-AD	**0.5 ±0.066**	0.440 ±0.084	N/A	N/S
		MAEO control	**0.37 ±0.095**	0.505 ±0.174	N/A	N/S
	Utianski 2016	PD-CN	0.41	0.59	N/A	N/A
		PD-D	0.43 [a]	0.55 [a]	N/A	N/A
		PD-MCI	0.42	0.56 [a]	N/A	N/A
		control	0.41	0.58	N/A	N/A
EEG upper alpha	*Das 2020*	EC MCI-AD	**0.491 ±0.062**	0.45 ±0.078	N/A	N/S
		EC control	**0.375 ± 0.079**	0.5 ±0.158	N/A	N/S
		EO MCI-AD	**0.55 ± 0.053**	0.44 ±0.174	N/A	N/S
		EO control	**0.37 ± 0.095**	0.505 ±0.084	N/A	N/S
		MAEC MCI-AD	**0.515 ± 0.078**	0.505 ±0.167	N/A	N/S
		MAEC control	**0.375 ± 0.079**	0.500 ±0.158	N/A	N/S
		MAEO MCI-AD	**0.475 ± 0.079**	0.44 ±0.046	N/A	N/S
		MAEO control	**0.37 ± 0.094**	0.505 ±0.174	N/A	N/S
	Utianski 2016	PD-CN	0.40	0.58	N/A	N/A
		PD-D	0.43 *a*	0.57	N/A	N/A

		PD-MCI	0.41	0.57	N/A	N/A
		healthy control	0.41	0.57	N/A	N/A
EEG beta	*Das 2020*	EC MCI-AD	**0.46 ± 0.077**	0.450 ±0.033	N/A	N/S
		EC control	**0.375 ± 0.079**	0.500 ±0.158	N/A	N/S
		EO MCI-AD	**0.46 ± 0.077**	0.430 ±0.026	N/A	N/S
		EO control	**0.37 ± 0.095**	0.505 ±0.174	N/A	N/S
		MAEC MCI-AD	**0.495 ± 0.0831**	0.460 ±0.046	N/A	N/S
		MAEC control	**0.375 ± 0.079**	0.500 ±0.158	N/A	N/S
		MAEO MCI-AD	**0.53 ± 0.042**	0.460 ±0.046	N/A	N/S
		MAEO control	**0.37 ± 0.095**	0.505 ±0.174	N/A	N/S
	Utianski 2016	PD-CN	0.41	0.56	N/A	N/A
		PD-D	0.41	0.57	N/A	N/A
		PD-MCI	0.42	0.56	N/A	N/A
		Healthy control	0.41	0.56	N/A	N/A
EEG gamma	*Das 2020*	EC MCI-AD	0.455 ±0.142	0.365 ± 0.129	N/A	N/S
		EC control	0.425 ±0.130	0.520 ± 0.211	N/A	N/S
		EO MCI-AD	0.455 ±0.142	0.360 ± 0.126	N/A	N/S
		EO control	0.415 ±0.123	0.440 ± 0.084	N/A	N/S
		MAEC MCI-AD	0.465 ±0.111	0.455 ± 0.174	N/A	N/S
		MAEC control	0.41 ±0.158	0.460 ± 0.097	N/A	N/S
		MAEO MCI-AD	0.455 ±0.142	0.360 ± 0.126	N/A	N/S
		MAEO control	0.41 ±0.158	0.490 ± 0.126	N/A	N/S
EEG	*Peraza 2018*	PDD	N/A	N/A	N/A	N/A
		DLB	N/A	N/A	N/A	N/A
		AD	N/A	N/A	N/A	N/A
		Healthy control	N/A	N/A	N/A	N/A
	Fraschini 2016	ALS	N/A*	N/A*	N/A*	N/A*
		Healthy control	N/A*	N/A*	N/A*	N/A*
MEG delta	*Sorrentino 2017*	AGHLS cohort	N/A	0.0515 ± 0.0148 [b]	N/A	0.3840 ± 0.0130

	Olde Dubbelink 2014	PD de novo	N/A	0.604 ± 0.023	N/A	0.437 ± 0.014
		Control	N/A	0.615 ± 0.037	N/A	0.450 ± 0.029
MEG theta	*Sorrentino 2017*	AGHLS cohort	N/A	0.5023 ± 0.0135	N/A	0.3777 ± 0.1309
MEG lower alpha	*Sorrentino 2017*	AGHLS cohort	N/A	0.5506 ± 0.0162	N/A	0.3795 ± 0.0157
MEG upper alpha	*Sorrentino 2017*	AGHLS cohort	N/A	0.5007 ± 0.0147	N/A	0.3758 ± 0.0139
	Olde Dubbelink 2014	PD de novo	N/A	**0.566 ± 0.018**	N/A	**0.423 ± 0.023**
		Healthy control	N/A	**0.594 ± 0.026**	N/A	**0.444 ± 0.023**
MEG beta	*Sorrentino 2017*	AGHLS cohort	N/A	0.5417 ± 0.0174 *b*	N/A	0.3740 ± 0.0154 *b*
MEG gamma	*Sorrentino 2017*	AGHLS cohort	N/A	0.4918 ± 0.0165	N/A	0.3708 ± 0.0138
MEG	*Jacini 2018*	Amnestic MCI	N/A	N/A	N/A*	N/A*
		Healthy control	N/A	N/A	N/A*	N/A*
	Lopez 2017	MCI	N/A*	N/A	N/A*	N/A*
		Healthy control	N/A*	N/A	N/A*	N/A*
	Sorrentino 2018	advanced ALS	N/A	N/A*	N/A	N/A*
		early ALS	N/A	N/A*	N/A	N/A*
		Healthy control	N/A	N/A*	N/A	N/A*
fMRI	*Wang 2018*	AD	N/A*	N/A*	N/A	N/A*
		MCI	N/A*	N/A*	N/A	N/A*
		Healthy control	N/A*	N/A*	N/A	N/A*
fMRI	*Saba 2019*	FTD	N/A	N/A	N/A	N/A
		Healthy control	N/A	N/A	N/A	N/A
DTI	*Jonak 2021*	LHON	0.192 ± 0.051	**0.533 ± 0.081**	N/A	**0.384 ± 0.056**
		Healthy control	0.174 ± 0.33	**0.603 ± 0.039**	N/A	**0.442 ± 0.035**

Data are presented as mean and when available SD. (SE) indicates the use of standard error instead of SD. Bold text represents significant results ($p < 0.05$). For EEG and MEG only frequency bands for which data was available are displayed.N/A: not available; measure not described in study, N/A* measure described in study; data not available, N/S: not significant, EEG: electroencephalography, fMRI: functional magnetic resonance imaging, DTI: diffusion tensor imaging, DLB: dementia with Lewy Bodies, AD: Alzheimer's disease, PD: Parkinson's disease, FTD: Frontotemporal dementia, SCD: subjective cognitive decline, MCI: mild cognitive impairment, EC: eyes closed EEG recording, EO: EO EEG recording, MAEO: mental arithmetic + eyes open EEG recording, MAEC: mental arithmetic + eyes closed EEG recording, ALS: amyotrophic lateral sclerosis, AGHLS: Amsterdam Growth and Health Longitudinal Study, LHON: Leber's hereditary optic neuropathy.

[a] significant compared to PD cognitively normal

[b] significant correlation with Insulin-like Growth Factor (IGF-1) levels

Supplementary table 5: MST metrics from studies on multiple sclerosis

Modality	First Author	Clinical group	Diameter	LF	Kappa	Th
MEG delta	*Tewarie 2015*	MS	0.222 ± 0.009	**0.543 ± 0.016**	N/A	0.411 ± 0.013
		Healthy control	0.218 ± (0.010	**0.556 ± 0.014**	N/A	0.416 ± 0.011
	Tewarie 2014 (a)	MS	N/A*	0.53 ± 0.022	3.1 ± 0.17	0.403 ± 0.023
		Healthy control	N/A*	0.53 ± 0.021	3.16 ± 0.17	0.403 ± 0.021
MEG theta	*Tewarie 2014 (b)*	MS	**0.18 (0.17–0.22)**	**0.62 (0.57–0.68)**	3.69 (3.17–4.36)	N/A (0.39–0.51)
		Healthy control	**0.21 (0.18–0.24)**	**0.59 (0.56–0.65)**	3.6 (3.14–4.44)	0.43 (0.40–0.47)
	Tewarie 2015	MS	**0.229 ± 0.010**	**0.541 ± 0.017**	N/A	0.413 ± 0.015
		Healthy control	**0.214 ± 0.006**	**0.555 ± 0.017**	N/A	0.416 ± 0.017
	Tewarie 2014 (a)	MS	N/A*	0.53 ± 0.024	3.10 ± 0.15	0.403 ± 0.023
		Healthy control	N/A*	0.53 ± 0.024	3.10 ± 0.19	0.405 ± 0.017
MEG lower alpha	*Tewarie 2015*	MS	0.229 ± 0.0105	0.544 ± 0.016 [a]	N/A	0.415 ± 0.013
		Healthy control	0.225 ± 0.010	0.555 ± 0.018 [a]	N/A	0.419 ± 0.013
MEG upper alpha	*Tewarie 2014 (b)*	MS	**0.21 (0.17–0.23)**	**0.59 (0.46–0.62)**	**3.54 (2.54–3.91)**	**0.43 (0.38–0.47)**
		Healthy control	**0.15 (0.12–0.21)**	**0.62 (0.58–0.68)**	**3.7 (3.39–4.92)**	**0.45 (0.42–0.52)**
	Tewarie 2015	MS	0.226 ± 0.010	0.542 ± 0.018	N/A	0.411 ± 0.014 [a]
		Healthy control	0.221 ± 0.013	0.556 ± .019	N/A	0.420 ± 0.011
	Tewarie 2014 (a)	MS	N/A*	**0.530 ± 0.021**	**3.1 ± 0.18**	**0.400 ± 0.019**
		Healthy control	N/A*	**0.540 ± 0.020**	**3.2 ± 0.14**	**0.410 ± 0.021**
MEG beta	*Tewarie 2014 (b)*	MS	0.22 (0.18–0.24)	0.58 (0.53–0.62)	3.33 (2.96–4.65)	0.43 (0.39–0.47)
		Healthy control	0.21 (0.19–0.22)	0.58 (0.56–0.61)	3.41 (3.12–4.32)	0.42 (0.39–0.45)
	Tewarie 2015	MS	0.225 ± 0.010	0.538 ± 0.017	N/A	0.420 ± 0.013
		Healthy control	0.221 ± 0.009	0.548 ± 0.015	N/A	0.414 ± 0.011
MEG gamma	*Tewarie 2015*	MS	0.225 ± 0.009	0.535± 0.018	N/A	0.404 ± 0.013
		Healthy control	0.221 ± 0.008	0.541 ± 0.015	N/A	0.408 ± 0.012
fMRI	*Tewarie 2015*	MS	0.320 ± 0.053	0.320 ± 0.053	N/A	0.270 ± 0.029 [a]
		Healthy control	0.320 ± 0.068	0.330 ± 0.030	N/A	0.260 ± 0.020 [a]

MEG	*Nauta 2020*	MS<br>follow up design	N/A*	N/A*	N/A	N/A*
			N/A*	N/A*	N/A	N/A*

Data is presented as mean and when available SD, except from Tewarie 2014 (b) where data is presented as median and range (min – max). Bold text represents significant results ($p < 0.05$). For MEG only frequency bands for which data was available are displayed.

N/A: not available; measure not described in study; N/A* measure described in study; data not available, EEG: electroencephalography, fMRI: functional magnetic resonance imaging, DTI: diffusion tensor imaging, MS: multiple sclerosis

[a] reported by Tewarie *et al.* as not significant anymore after correction for multiple comparisons.

Supplementary table 6: MST metrics from studies on epilepsy

6a

**Modality**	**First author**	**Clinical group**	**Diameter**	**LF**	**Kappa**	**Th**
EEG delta	*Van Diessen* 2016	Chld GE	0.474 ± 0.009 (SE)	0.539 ± 0.009 (SE)	N/A	N/A
		Chld FE	0.490 ± 0.006 (SE)	**0.528 ± 0.006 (SE)**	N/A	N/A
		Healthy Control	0.466 ± 0.003 (SE)	0.547 ± 0.003 (SE)	N/A	N/A
EEG theta	*Van Diessen 2016*	Chld GE	0.471 ± 0.00 (SE)	0.545 ± 0.009 (SE)	N/A	N/A
		Chld FE	0.471 ± 0.006 (SE)	0.547 ± 0.006 (SE)	N/A	N/A
		Healthy Control	0.473 ± 0.003 (SE)	0.548 ± 0.004 (SE)	N/A	N/A
EEG lower alpha	*Van Diessen 2016*	Chld GE	0.464 ± 0.00 (SE)	0.562 ± 0.010 (SE)	N/A	N/A
		Chld FE	**0.456 ± 0.006 (SE)**	**0.569 ± 0.007 (SE)**	N/A	N/A
		Healthy Control	0.479 ± 0.004 (SE)	0.545 ± 0.004 (SE)	N/A	N/A
EEG upper alpha	*Van Diessen 2016*	Chld GE	0.459 ± 0.010 (SE)	0.578 ± 0.011 (SE)	N/A	N/A
		Chld FE	0.454 ± 0.007 (SE)	0.576 ± 0.008 (SE)	N/A	N/A
		Healthy Control	0.458 ± 0.004 (SE)	0.569 ± 0.004 (SE)	N/A	N/A
EEG beta	*Van Diessen 2016*	Chld GE	0.502 ± 0.008 (SE)	0.516 ± 0.009 (SE)	N/A	N/A
		Chld FE	0.494 ± 0.005 (SE)	0.516 ± 0.006 (SE)	N/A	N/A
		Healthy Control	0.494 ± 0.003 (SE)	0.523 ± 0.003 (SE)	N/A	N/A
EEG	*Van Diesen 2014*	Chld. suspected new onset Focal epilepsy	N/A*	N/A*	N/A	N/A
		Healthy control (sleep deprivation design)	N/A*	N/A*	N/A	N/A

6b

Modality	First author	Clinical group	Diameter	LF	Kappa	Th
EEG theta	*Fraschini 2014*	Nonresponder pre-VNS	**0.404 ± 0.023**	0.606 ± 0.034	N/A	0.407 ± 0.030
		Nonresponder post-VNS	**0.463± 0.029**	0.544 ± 0.064	N/A	0.380 ± 0.053
		Responder pre-VNS	**0.433 ± 0.046**	0.581 ± 0.081	N/A	0.396 ± 0.064
		Responder post-VNS	**0.359 ± 0.045**	0.667 ± 0.067	N/A	0.442 ± 0.036
MEG alpha	*Van Dellen 2014*	Meningioma, with seizures				
		Post operative seizures (baseline)	0.2069 ± 0.0148	0.5907 ± 0.0180	3,5377 ± 0,1818	N/A
		Post operative seizure free (baseline)	0.2198 ± 0.0241	0.5673 ± 0.033	3,3435 ± 0,3044	N/A
		Post operative seizures (9-15 month follow up)	0.2139 ± 0.0170	0.5859 ± 0.0226 $^{\$}$	3,6055 ± 0,3647	N/A
		Post operative seizure free (9-15 month follow up)	0.2092 ± 0.0228	0,5932 ± 0,0383 $^{\$}$	3,5417 ± 0,3882	N/A
fMRI	*DeSalvo 2020*	TLE pre-op, seiz post-op	N/A	N/A*	N/A	N/A*
		TLE pre-op, seiz free post-op (n=22)	N/A	N/A*	N/A	N/A*

6c

Modality	First author	Clinical group	Diameter	LF	Kappa	Th
EEG	*Kinney-Lang 2019*	Chld early onset epilepsy				
		Cognitively normal	N/A*	N/A*	N/A	N/A
		Mild cognitive impairment (n=7)	N/A*	N/A*	N/A	N/A
		Severe cognitive impairment (n=13)	N/A*	N/A*	N/A	N/A

Segment *a* represents studies assessing disease effect. Segment *b* represents studies assessing treatment effect. Segment *c* contains one study assessing the correlation between MST metrics and cognitive scores.

Data are presented as mean and when available SD. Bold text represents significant results ($p < 0.05$). For EEG and MEG only frequency bands for which data was available are displayed. N/A: not available; measure not described in study, N/A* measure described in study; data not available, EEG: electroencephalography, fMRI: functional magnetic resonance imaging, Chld GE: Drug naive children with generalized epilepsy, Chld FE: Drug naive children with focal epilepsy, TLE: temporal lobe epilepsy, VNS: Vagal nerve stimulation.

$^{\$}$: significant difference compared to seizure free group at 9-15 months after surgery.

Supplementary table 7: MST metrics from studies on other disorders

Modality	First Author	Clinical group	Diameter	LF	Kappa	Th
EEG delta	*Numan 2017*	Hypo-active delirium	0.481 (0.449-0.493)	0.535 (0.504-0.579)	N/A	0.376 (0.355-0.408)
		Recovery anesthesia	**0.457 (0.438-0.469)**	**0.558 (0.532-0.594)**	N/A	0.385 (0.366-0.402)
		Non-delirious control	0.486 (0.446-0.516)	0.522 (0.500-0.563)	N/A	0.371 (0.356-0.388)
EEG alpha	*Numan 2017*	Hypo-active delirium	0.497 (0.454-0.516	**0.513 (0.485-0.547)**	N/A	0.370 (0.350-0.398)
		Recovery anesthesia	0.467 (0.442-0.496)	0.534 (0.493-0.563)	N/A	0.368 (0.346-0.394)
		Non-delirious control	0.469 (0.438-0.500)	0.545 (0.516-0.574)	N/A	0.377 (0.363-0.393
EEG beta	*Numan 2017*	Hypo-active delirium	0.522 (0.485-0.563)	0.485 (0.450-0.531)	N/A	0.351 (0.330-0.370)
		Recovery anesthesia	0.512 (0.485-0.547)	0.511 (0.488-0.532)	N/A	0.372 (0.360-0.389)
		Non-delirious control	0.510 (0.485-0.543)	0.501 (0.469-0.532)	N/A	0.360 (0.343-0.382)
MEG	*Nieboer 2020*	Migraine	N/A*	N/A*	N/A*	N/A*
		subgroup recently diagnosed	N/A*	N/A*	N/A*	N/A*
		subgroup long-term	N/A*	N/A*	N/A*	N/A*
		Healthy controls	N/A*	N/A*	N/A*	N/A*
MEG	*Van Nieuwenhuizen 2018*	meningioma patients	N/A	N/A*	N/A	N/A*
		Healthy controls	N/A	N/A*	N/A	N/A*
fMRI	*Van Montfort 2018*	Delirium	**0.30 ± 0.05**	**0.32 ± 0.04**	2.38 ± 0.11	0.26 ± 0.02
		Post Delirium	0.30 ± 0.06	0.36 ± 0.04	2.45 ± 0.11	0.31 ± 0.03
		Non delirious control	0.28 ± 0.04	0.35 ± 0.03	2.41± 0.07	0.28 ± 0.02

Data is presented as mean and when available SD. For Numan *et al* data is presented as median and Interquartile range. Bold text represents significant results ($p < 0.05$). For EEG and MEG only frequency bands for which data was available are displayed. For EEG and MEG only frequency bands for which data was available are displayed. N/A: not available; measure not described in study, N/A* measure described in study; data not available, NS: not significant, EEG: electroencephalography, fMRI: functional magnetic resonance imaging.
